# Antibody to *P. falciparum* in Pregnancy Varies with Intermittent Preventive Treatment Regime and Bed Net Use

**DOI:** 10.1371/journal.pone.0029874

**Published:** 2012-01-27

**Authors:** Elizabeth H. Aitken, Bernard Mbewe, Mari Luntamo, Teija Kulmala, James G. Beeson, Per Ashorn, Stephen J. Rogerson

**Affiliations:** 1 Department of Medicine (RMH/WH), University of Melbourne, Parkville, Victoria, Australia; 2 Department of Community Health, College of Medicine, University of Malawi, Blantyre, Malawi; 3 Department of International Health, School of Medicine, University of Tampere, Tampere, Finland; 4 Burnet Institute, Melbourne, Victoria, Australia; 5 Department of Paediatrics, Tampere University Hospital, Tampere, Finland; Université Pierre et Marie Curie, France

## Abstract

**Background:**

Antibodies towards placental-binding *P. falciparum* are thought to protect against pregnancy malaria; however, environmental factors may affect antibody development. Methods and Findings: Using plasma from pregnant Malawian women, we measured IgG against placental-binding *P. falciparum* parasites by flow cytometry, and related results to intermittent preventive treatment (IPTp) regime, and bed net use. Bed net use was associated with decreased antibody levels at mid-pregnancy but not at 1 month post partum (1 mpp). At 1 mpp a more intensive IPTp regime was associated with decreased antibody levels in primigravidae, but not multigravidae.

**Conclusions/Significance:**

Results suggest bed nets and IPTp regime influence acquisition of pregnancy-specific *P. falciparum* immunity.

## Introduction

*Plasmodium falciparum* parasites that accumulate in the placenta express unique variant surface antigens (VSA) including the protein VAR2CSA, which enables the parasitised erythrocyte to adhere to placental chondroitin sulphate-A (CSA) [1]. IgG antibodies towards CSA-VSA (CSA-binding phenotype VSAs) are gravidity dependent and have been associated with protection from malaria-related low birth weight and maternal anaemia [1]. The development of these antibodies may depend on a combination of factors, including maternal genetics, HIV status and parasite exposure, which in turn could vary with the use of intermittent preventive treatment in pregnancy (IPTp) and bed nets. Published data suggests IPTp may decrease CSA-VSA IgG acquisition [2], [3] and that this may be dependent on the presence of HIV infection [2]. The effect of bed net use on development of these antibodies is unknown. Antibody development may be further affected by changes in transmission intensity that decrease exposure to the antigen; and declines in pregnancy malaria prevalence have been recently reported [4]. To examine the effect of bed nets and IPTp on CSA-VSA IgG levels we measured antibody towards a CSA-binding parasite line during pregnancy and post partum in pregnant Malawian women participating in a randomised clinical trial of different IPTp regimes.

## Materials and Methods

### Study population, sample and clinical data collection

The study population was a consecutive subset of women (selected based on sample availability) from the Lungwena Antenatal Intervention Study (LAIS) cohort (NIH registration NCT00131235). Following written informed consent, pregnant women at 14–26 gestation weeks (gw) were enrolled at an antenatal clinic in Lungwena, Malawi, between December 2003 and October 2006. Women were randomly allocated to one of three groups for IPTp treatment: sulfadoxine-pyrimethamine (SP) (1500 mg/75 mg) twice during pregnancy; monthly SP from enrolment, finishing before 37 gw; or azithromycin (AZI, 1000 mg) twice during pregnancy and monthly SP.

Parasitemia prevalence at enrolment and 28–34 gw was determined by microscopy of Giemsa stained peripheral blood smears. Maternal HIV status at enrolment was determined using two rapid diagnostic tests (Determine, Abbot Laboratories, USA and Uni-Gold, Trinity Biotech, Ireland). Bed net use, socioeconomic status (maternal literacy and schooling) and gravidity were determined using a questionnaire at enrolment. Serum separated from venous or finger prick blood samples taken at enrolment and 1-month post partum (mpp) was frozen and shipped to Melbourne for IgG measurement and analysis. Ethical approval was provided by the Human Research Ethics Committee, Walter and Eliza Hall Institute of Medical Research, Ethical Committee of Pirkanmaa Hospital District in Finland, and the College of Medicine Research and Ethics Committee, University of Malawi.

### P. falciparum line

Parasitised red blood cells (PRBC) infected with *P. falciparum* line CS2, which binds CSA and expresses *var2csa*, were cultured [5] in RPMI-HEPES medium with 0.2% w/vol NaHCO3 and 0.5% Albumax II (GIBCO™) in Group-O red blood cells (RBC)(Australian Red Cross Blood Service).

### Measurement of IgG

Flow cytometry was used to measure IgG levels to VSA on the PRBC (CSA-VSA IgG), as described previously [5]. Heat inactivated human sera (1∶20 dilution; in duplicate) were co-incubated with 4–8% trophozoite stage PRBC at 0.1% haematocrit in PBS with 1% neonatal calf serum, with polyclonal rabbit anti-human IgG antibody (1∶100, DakoCytomation, Denmark) and with Alexafluor® 488 donkey anti-rabbit IgG (1∶500, Invitrogen, USA) with 10 µg/ml ethidium bromide (EtBr). A relative geometric mean fluorescence intensity (relative MFI) for PRBC was calculated using negative (unexposed adults) and positive (pooled serum with high CSA-VSA IgG) controls.

### Data analysis

Data were analysed using Stata (Version9, Stata Corp., USA). CSA-VSA IgG levels (relative MFI) were log transformed prior to regression analysis. Unless otherwise stated results are for multivariate analysis with relative MFI at enrolment and 1 mpp defined as outcome variables, and bed net use, IPTp regime, HIV status, gravidity (primi-, secundi- or multigravidae) and parasitemia defined as exposure variables. P values of <0.05 were taken as significant.

## Results

CSA-VSA IgG was measured in 538 women at enrolment (14–26 gw) and 364 women at 1 mpp; 355 women had samples assayed at both time points. CSA-VSA IgG at enrolment and 1 mpp was positively associated with gravidity (Table 1). The mean (SD) age of the participants was 24.9 (6.7) years. Neither participants' age, gravidity, HIV nor socioeconomic status varied significantly with bed net use or IPTp regime; bed net use and ITN regime were also not associated with each other.

**Table 1:** Association between specified factors and IgG to CS2-VSA, multivariate analysis.

			Enrolment		1 month post partum		
		n (%)	Co-efficient (95%CI)	P	n (%)	Co-efficient (95%CI)	P
Gravidity	primigravidae	129 (24.0)			81 (22.3)		
	secundigravidae	107 (19.9)	0.402 (−0.014, 0.818)	0.058	77 (21.2)	0.805 (0.139, 1.472)	0.018
	multigravidae	302 (56.1)	1.335 (0.996, 1.673)	<0.001	206 (56.6)	1.627 (1.063, 2.191)	<0.001
HIV^1^	negative	400 (74.3)			279 (76.6)		
	positive	66 (12.3)	−0.395 (−0.783, −0.006)	0.047	35 (9.6)	−0.291 (−0.988, 0.405)	0.41
	unknown	72 (13.4)			50 (13.7)		
ITN^1^	negative	200 (37.2)			139 (38.2)		
	positive	338 (62.8)	−0.449 (−0.728, −0.171)	0.002	225 (61.8)	−0.251 (−0.699, 0.197)	0.27
Parasitemia^2^	negative	492 (91.5)			331 (90.9)		
	positive	46 (8.6)	0.469 (−0.019, 0.957)	0.060	33 (9.1)	−0.391 (−1.159, 0.362)	0.30
Treatment^3^	Two-dose SP				118 (32.6)		
	monthly SP				115 (31.6)	−0.352 (−0.889, 0.184)	0.20
	monthly-SP+AZI				131 (36.0)	−0.366 (−0.882, 0.150)	0.16

At enrolment, bed net use (prevalence 63%) and HIV infection (prevalence 14%) were associated with lower CSA-VSA IgG. Concurrent *P. falciparum* parasitemia (prevalence 9%) was associated with a non-significantly higher CSA-VSA IgG level (Table 1). When the data were stratified by gravidity, there was a significant negative association between bed net use and CSA-VSA IgG at enrolment in primigravidae (coefficient −0.672; 95%CI −1.245, −0.099, P = 0.022), but not in multigravidae (coefficient −0.307; 95% CI −0.632, 0.018 P = 0.064) or secundigravidae (coefficient −0.588; 95% CI −1.442, 0.266 P = 0.18). HIV infection was associated with non-significantly lower CSA-VSA IgG at enrolment in primigravidae (coefficient;−0.709 95% CI −1.869, 0.276 P = 0.14) and multigravidae (coefficient −0.404; 95% −0.826, 0.016 P = 0.059) but not secundigravidae (coefficient −0.060; 95% −1.237, 1.118 P = 0.92).

**Figure 1:**
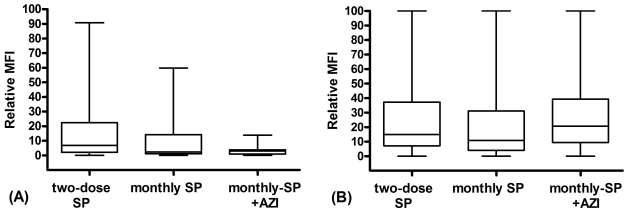
Relationship between antibody to CS2 variant surface antigens and IPTp regime, stratified by gravidity.

At 1 mpp, among all women tested, levels of CSA-VSA IgG were non-significantly lower in women who received SP+AZI compared to women who received two-dose SP (Table 1), and CSA-VSA IgG levels in women who received monthly SP were intermediate (compared to the other two regimes). When stratified by gravidity, there were significantly lower levels in primigravidae who received SP+AZI than those who received two-dose SP (coefficient −1.447; 95% CI −2.800, −0.094 P = 0.036); levels in primigravidae who received monthly SP were intermediate (coefficient −1.189; 95% CI −2.613, 0.233 P = 0.1) (Figure 1A). Secundigravidae who received SP+AZI had non-significantly lower levels compared two-dose SP (coefficient −0.989; 95% CI −2.213, 0.235 P = 0.11); and similar levels to secundigravidae who received monthly SP (coefficient −0.371; 95% CI −1.634, 0.893 P = 0.56). In contrast to primigravidae, multigravidae who received two-dose SP had similar CSA-VSA IgG levels to those who had monthly SP (coefficient −0.088; 95% CI −0.729, 0.552 P = 0.79) and to those who had received SP+AZI (coefficient 0.118; 95% CI −0.495, 0.731 P = 0.71) (Figure 1B).

Because HIV infection has been associated with decreased antibody response to IPTp, we assessed whether the relationships between both IPTp and bed net use and antibody development were also seen in HIV negative women. Among HIV negative women who received SP+AZI, only primigravidae had significantly lower CSA-VSA IgG than those who received two-dose SP (coefficient-1.400; 95%CI −2.769- −0.032, P = 0.045). HIV negative women who used bed nets had significantly lower CSA-VSA IgG at enrolment compared to those who did not use bed nets (coefficient −0.501; 95%CI −0.802, −0.200, P = 0.001), and this did not appear to be explained by differences in schooling or literacy (data not shown). Due to the low HIV prevalence, HIV positive women were not separately assessed for the effects of IPTp regime or bed net use on antibody levels.

## Discussion

We examined the association between CSA-VSA IgG antibodies and use of bed nets and IPTp in the context of a randomised clinical trial in pregnant women. We found that bed net use and intensive IPTp regimes were associated with lower levels of these antibodies. These associations were not dependent on the presence of HIV infection, but the association between IPTp regime and antibody was clearly dependent on gravidity. At antenatal booking, antibody levels were lower in women who used bed nets or who were HIV positive. At 1 mpp the most intensive IPTp regime was associated with decreased antibody in primigravidae. The observation that the difference was between the SP+AZI and two-dose SP regimes, not two-dose SP and monthly SP regimes, suggests that it is the drug AZI which accounts for the lower CSA-VSA IgG levels. Interestingly, a high frequency (90%) of mutations associated with SP resistance has been found in *P. falciparum* infections in Malawi [6].

That bed net use is associated with decreased antibody is a novel finding, and may indicate that bed nets decreased exposure to CSA-binding parasites before antenatal clinic attendance. Whereas most antimalarial drugs are not recommended in the first trimester, using bed nets from conception may decrease parasite exposure in early pregnancy. We found that bed net use was not associated with antibody at 1 mpp, while the monthly SP+AZI regime was, suggesting that intensive IPTp, which may both clear existing infection and inhibit new infection, may be a more important determinant than bed net use of the development of antibody in the latter part of pregnancy.

Previous studies have yielded conflicting findings on the relationship between IPTp and pregnancy-specific immunity [2], [3]. Staalsoe et al showed that CSA-VSA IgG levels were decreased among primigravidae receiving SP over placebo. In Mozambique, women of all gravidities did not show decreased CSA-VSA IgG with two doses of SP-IPTp, unless they were HIV positive [2]. HIV infected women may require more than two doses of SP to be protected from pregnancy malaria [7]. In the present study, the association between IPTp and antibody development was not restricted to HIV positive women. Differences between studies may be due to variation in SP resistance, both Mozambique and Malawi have SP resistant parasites [8], [9]. Given the recommendation that all pregnant women in areas like Lungwena receive IPTp [10], we had no placebo group. Instead, we observed a clear relationship between more intensive IPTp regimes and antibody levels in primigravidae, suggesting that the more intensive regimes decrease exposure to the antigen, decreasing antibody development. In keeping with this, we observed a lower prevalence of *P. falciparum* infection (detected by microscopy of Giemsa stained peripheral blood smears) at 32 weeks and at delivery in women receiving monthly SP ± AZI (2% at each time point) than in women receiving two-dose SP (5% at each time point) [6]. Parasite prevalence at delivery estimated by quantitative PCR was also significantly higher in women receiving two-dose SP (Luntamo et al, in preparation).

The observation that IPTp use was only associated with antibody levels in primigravidae may reflect higher existing levels of immunity in multigravidae, such that decreased antigen exposure with intensive IPTp regimes had minimal effects on immunity. The protective effect of IPTp may also be more modest in women with existing immunity, and these women, usually multigravidae, may be more capable of boosting antibody levels [5] in response to sub microscopic infections, which were relatively common [11].

At enrolment, history of bed net use was associated with decreased antibody in both primigravidae and multigravidae, although the association only reached statistical significance in primigravidae. By contrast, IPTp regime was only associated with antibody level at delivery in primigravidae. This difference in associations between bed nets and IPTp with antibody may be due to the total time that the intervention can decrease antigen exposure (as bed net use might reflect decreased exposure over a prolonged period, not just the current pregnancy), or to the ability of bed nets to block boosting of antibodies by infection in early pregnancy.

Our study had a number of potential shortcomings. The low prevalence of HIV and parasitemia compromised our ability to identify relationships between these factors and antibody. We only measured antibody at two time points during pregnancy, but antibody levels may rapidly decrease following clearance of infection, and are in any case highly dynamic [5]. Being a community based study, it was usually not possible to document placental infection and as peripheral parasitemia was measured by microscopy [11] and at limited time points, parasitaemia prevalence was likely underestimated. (Only a subset of women in the present study also contributed to the comparison of qPCR and microscopy). In choosing samples from a sequential set of women for this study, the probability of selection bias was minimised, and the LAIS used population based sampling [6]. Socioeconomic factors that could indicate altered risk of *P. falciparum* exposure, potentially compromising analyses, were not associated with HIV or bed net use. We could, of course, only determine statistical associations, not causal relationships, between interventions and antibody development.

If bed nets and intensive IPTp decrease pregnancy-specific immunity, this may have important implications for the ongoing susceptibility of pregnant women to malaria. As coverage with bed nets and IPTp rises, more women may receive their benefits, but they may be increasingly vulnerable in subsequent pregnancies. The only study that examined this found that chemoprophylaxis in the third trimester did not affect malaria susceptibility in the subsequent pregnancy, but participants may have already experienced substantial exposure and developed immunity by this time [12]. If bed nets and IPTp cease to be widely available, many highly-susceptible pregnant women could be again exposed to high risk of pregnancy malaria, with potentially devastating consequences. Whether the relationships between bed nets and IPTp use and development of immunity are similar in higher transmission settings, is currently unknown. The effects of decreased exposure and decreased antibody on susceptibility to disease need to be properly addressed by longitudinal studies over successive pregnancies.

## References

[1] Rogerson SJ, Hviid L, Duffy PE, Leke R, Taylor DW (2007). Malaria in pregnancy: pathogenesis and immunity.. Lancet.

[2] Serra-Casas E, Menendez C, Bardaji A, Quintó L, Dobaño C (2010). The effect of intermittent preventive treatment during pregnancy on malarial antibodies depends on HIV status and is not associated with poor delivery outcomes.. J Infect Dis.

[3] Staalsoe T, Shulman CE, Dorman EK, Kawuondo K, Marsh K (2004). Intermittent preventive sulfadoxine-pyrimethamine treatment of primigravidae reduces levels of plasma immunoglobulin G, which protects against pregnancy-associated *Plasmodium falciparum* malaria.. Infect Immun.

[4] Feng G, Simpson JA, Chaluluka E, Molyneux ME, Rogerson SJ (2010). Decreasing burden of malaria in pregnancy in Malawian women and its relationship to use of intermittent preventive therapy or bed nets.. PLoS One.

[5] Aitken EH, Mbewe B, Luntamo M, Maleta K, Kulmala T (2010). Antibodies to chondroitin sulfate A-binding infected erythrocytes: dynamics and protection during pregnancy in women receiving intermittent preventive treatment.. J Infect Dis.

[6] Luntamo M, Kulmala T, Mbewe B, Cheung YB, Maleta K (2010). Effect of repeated treatment of pregnant women with sulfadoxine-pyrimethamine and azithromycin on preterm delivery in Malawi: A randomized controlled trial.. Am J Trop Med Hyg.

[7] van Eijk AM, Ayisi JG, ter Kuile FO, Otieno JA, Misore AO (2004). Effectiveness of intermittent preventive treatment with sulphadoxine-pyrimethamine for control of malaria in pregnancy in western Kenya: a hospital-based study.. Trop Med Int Health.

[8] Enosse S, Magnussen P, Abacassamo F, Gómez-Olivé X, Rønn AM (2008). Rapid increase of Plasmodium falciparum dhfr/dhps resistant haplotypes, after the adoption of sulphadoxine-pyrimethamine as first line treatment in 2002, in southern Mozambique.. Malar J.

[9] Nkhoma S, Molyneux M, Ward S (2007). Molecular surveillance for drug-resistant Plasmodiumfalciparum malaria in Malawi.. Acta Trop.

[10] WHO (2004). A strategic framework for malaria prevention and control during pregnancy in the African region.

[11] Rantala AM, Taylor SM, Trottman PA, Luntamo M, Mbewe B (2010). Comparison of real-time PCR and microscopy for malaria parasite detection in Malawian pregnant women.. Malar J.

[12] Greenwood AM, Menendez C, Alonso PL, Jaffar S, Langerock P (1994). Can malaria chemoprophylaxis be restricted to first pregnancies?. Trans R Soc Trop Med Hyg.

